# MUC3A promotes the progression of colorectal cancer through the PI3K/Akt/mTOR pathway

**DOI:** 10.1186/s12885-022-09709-8

**Published:** 2022-06-02

**Authors:** Wei Su, Baijie Feng, Lina Hu, Xianzhi Guo, Minghua Yu

**Affiliations:** Department of Oncology, Fudan University Pudong Medical Center, Shanghai Pudong Hospital, 2800 Gongwei Road, Shanghai, 201399 China

**Keywords:** MUC3A, Colorectal cancer, PI3K, Akt, mTOR pathway, Cell cycle, Cancer progression

## Abstract

Mucin 3A (MUC3A) is overexpressed in colorectal cancer (CRC) and associated with poor prognosis, but the related mechanism remains unclear. Our study found that MUC3A promotes the progression of CRC by activating the PI3K/Akt/mTOR signaling pathway. Knockout of MUC3A significantly inhibited the proliferation of CRC cells and induced G1 phase arrest by upregulating p21 protein, an important cell cycle regulator. Moreover, knockout of MUC3A significantly inhibited invasion ability and enhanced the sensitivity to the chemotherapeutic agent 5-FU. Furthermore, we found that knockout of MUC3A repressed the PI3K/Akt/mTOR pathway through RNA-seq. Treatment with the PI3K/Akt/mTOR pathway inhibitor rapamycin successfully eliminated the difference in proliferation, invasion and chemoresistance between MUC3A knockout cells and control cells. Our study suggests that MUC3A is a potential oncogene that promotes the proliferation, invasion, and chemotherapy resistance of CRC. Moreover, CRC patients with high expression of MUC3A may benefit from rapamycin treatment.

**Supplementary Information:**

The online version contains supplementary material available at 10.1186/s12885-022-09709-8.

## Introduction

CRC is a common gastrointestinal malignancy, with approximately 1.88 million new cases worldwide in 2020 alone, accounting for 10% of all types of tumors [1]. Surgery in early-stage CRC can achieve a 90% cure rate but the rate is less than 20% in advanced CRC [2]. For these patients, surgery may not be possible, and the severe side effects of radiation and chemotherapy are often the main challenges [3, 4]. In chemotherapy regimens for colorectal cancer, continuous 5-Fluorouracil (5-FU) infusion regimens, such as FOLFOX or FOLFIRI, have been established and are widely utilized [5]. 5-FU is a key anticancer drug, which has been used to treat various types of malignant tumors due to its extensive antitumor activity and synergistic effect with other anticancer drugs. However, the severe side effects and drug resistance of 5-FU are very common. Therefore, it is necessary and urgent to explore new oncogenes in the development of CRC that can be applied as early diagnostic markers and therapeutic targets.

MUC3A, a main member of the mucin family, is commonly expressed on the surface of various epithelial cells, especially intestinal epithelial cells. MUC3A contains a sperm protein, enterokinase, agrin, and epithelial growth factor (EGF) domain and functions through ligand binding and intracellular signaling pathways [6]. In normal tissues as well as inflammatory diseases, such as reflux esophagitis, ulcerative colitis and Crohn's disease, MUC3A promotes cell migration, inhibits apoptosis and accelerates wound healing [7–10]. Interestingly, MUC3A is overexpressed in various tumors, including renal, gastric, non-small cell lung and CRC, and is often associated with metastasis and poor prognosis [11–14]. In the currently available studies, MUC3A decreased the sensitivity of radiotherapy for non-small cell lung cancer [15] and reduced the efficacy of targeted therapy by inducing PD-L1 [13]. MUC3A is also an effective antigenic target for CAR-T cell therapy in gastric adenocarcinoma [12]. However, few studies have investigated the role of MUC3A in CRC, and the relevant mechanism remains unclear. Therefore, our research aimed to investigate the role of MUC3A in CRC and the possibility of MUC3A as a new therapeutic target in CRC.

## Materials and methods

### Cell culture and transfection

The human colon cancer cell lines HCT116 and SW620 were obtained from the University of Colorado Cancer Center Cell Bank. HCT116 cells were cultured in McCoy’s 5A medium supplemented with 10% FBS (Invitrogen, Carlsbad, CA, USA) at 37 °C in a 5% CO_2_ atmosphere. SW620 cells were cultured in L-15 medium supplemented with 10% FBS (Invitrogen, Carlsbad, CA, USA) at 37 °C in a 100% air atmosphere.

The sgRNAs (KO1: GAAGGCAAACTTGGACGTCG; KO2: TTCTCCACACTCCAGAGACC) of human MUC3A were synthesized by Genomeditech, Inc. (Shanghai, China) and cloned into the lentiCRISPR v2 lentiviral vector to construct the lentiCRISPR-MUC3A knockout plasmids. Based on the instructions of the product manual, Lipofectamine 3000 (Invitrogen, Inc.) was used to cotransfect the target plasmid or the scrambled vector, psPAX2, and PMG.2G into HEK293T tool cells to obtain MUC3A knockout lentivirus or scrambled control lentivirus. Then, the lentivirus (multiplicity of infection, MOI = 10) was used to infect HCT116 and SW620 cells. The MUC3A knockout cell lines HCT116-KO1, KO2, SW620-KO1, and KO2 and the negative control cell lines HCT116-NC and SW620-NC were screened by puromycin (2 μg/mL, 72 h). The knockout of MUC3A was confirmed by Western blot. Western blot assays verified that MUC3A protein expression was significantly reduced in HCT116 cells after MUC3A gene knockout.

### Western blotting analysis

Total cellular proteins from each group were extracted using RIPA lysis buffer with protease inhibitor cocktail and phosphatase inhibitor cocktail. Then, equal amounts (20 μg) of protein determined by a BCA protein assay kit (Thermo Fisher Scientific, USA) were separated using 6%-10% SDS–PAGE gels. The proteins were then transferred to PVDF membranes (0.45 mm, Solarbio, China) at 300 mA for 2 h. And the blots were cut prior to binding with antibodies during blotting. The membranes were blocked with 5% non-fat milk in TBST for 1 h at room temperature and then incubated overnight with primary antibodies at 4 °C. The following antibodies were tested: MUC3A polyclonal antibody (#PA5-95,355, 1:1000, Thermo Fisher Scientific, MA, USA), p-PI3K (#4228), PI3K (#4257), p-Akt (#4060), Akt (#468), p-mTOR (#5536), mTOR (#2983), p53 (#2527) rabbit monoclonal antibodies (1:1000, CST, MA, USA), and p21 (#A19094) and β-tubulin (#A17913) rabbit monoclonal antibodies (1:1000, Abclonal, Wuhan, China). The secondary antibodies were anti-mouse or anti-rabbit antibodies and were conjugated to horseradish peroxidase (HRP) (1:4000, Abclonal, Wuhan, China). The antibodies were used at a 1:4000 dilution and incubated at room temperature for approximately 1 h. The bands were visualized with ECL reagents (Thermo Fisher Scientific, MA, USA) and developed by Omega Lum G (Aplegen, USA).

### RNA extraction, reverse transcription and quantitative PCR (RT–qPCR)

Total RNA was extracted from cells by TRIzol reagent (Invitrogen). cDNA was obtained from total RNA using a PrimeScript™ RT reagent kit (Takara Bio, Inc., Otsu, Japan). mRNA expression was assessed by real-time quantitative PCR with a SYBR Premix Ex Taq™ kit (Takara Bio) and ABI 7900HT Real-Time PCR system (Applied Biosystems Life Technologies, CA, USA) were performed in triplicate. Comparative cycle threshold values (2^−ΔΔCt^) were applied to analyze the final results.

### RNA sequencing (RNA-seq)

RNA-seq was carried out at Jiayin Biotechnology Ltd. (Shanghai, China). Sequencing libraries were generated using NEBNext UltraTM RNA Library Prep Kit for Illumina (NEB, MA, USA) following manufacturer’s recommendation and index codes were added to attribute sequences to each sample. The fragment sizes were 250–300 bps. The clustering of the index-coded samples was performed on a cBot Cluster Generation System using TruSeq PE Cluster Kit v3-cBot-HS (Illumina, CA, USA) according to the manufacturer’s instructions. After cluster generation, the library preparations were sequenced on an Illumina Novaseq6000 platform and 150 bp paired-end reads were generated. HTSeq v0.6.0 was used to count the reads numbers mapped to each gene. We applied DESeq2 algorithm to filter the differentially expressed genes. The statistical power of this experimental design, calculated in differential expression analysis is the following criteria: i) |log2FC|> 1; ii) *P*-value < 0.05. Pathway analysis was used to find out the significant pathway of the differential genes according to KEGG database. The statistical power of this experimental design, calculated in the significant pathway of the differential genes is clusterProfiler R package. We turn to the Fisher’s exact test to select the significant pathway, and the threshold of significance was defined by *P*-value and FDR.

### Cell migration and invasion assays

Cell migration and invasion were performed by Transwell plates (24-well insert, 8 μm pore size; BD Biosciences, Bedford, MA, USA). The filters (Corning, USA) were coated with (invasion) or without (migration) 55 μl Matrigel (1:8 dilution; BD Biosciences). For the migration assays, 1 × 10^5^ cells were suspended in 200 μl serum-free medium and seeded into upper chambers that were not coated with Matrigel. Then, 500 μl of medium containing 10% FBS was added to the lower chamber acting as a chemoattractant. After incubation at 37 °C for 48 h, the membranes were fixed with 4% formaldehyde for 30 min and stained with 0.1% crystal violet for 30 min at room temperature. For invasion assays, 1 × 10^5^ cells suspended in 200 μl serum-free medium were seeded into Matrigel-coated upper chambers. The rest of the protocol was identical to that described above. The cells were counted and photographed under an inverted microscope in 5 different areas of each triplicate filter [16].

### Wound healing assay

For this assay, 8 × 10^5^ cells were seeded into 6-well plates and incubated at 37 °C for 24 h. We used a 200 μl sterile micropipette tip to scratch a straight line through the confluent monolayers when cells were 80–90% confluent. We then washed floating cells with PBS three times and continued to incubate the cells after changing the complete medium to serum-free medium. Images of the same wound position were taken under the microscope at 0 h and 24 h. The migration results were tested by ImageJ software [16].

### Cell proliferation assay

For this assay, 5 × 10^3^ cells were seeded into 96-well plates and cultured for the following times: 0 h, 24 h, 48 h and 72 h. Before the assay, 10 µl of Cell-Counting Kit-8 (CCK-8; Dojindo Molecular Technologies, Japan) solution was added to each well of the plate, and incubated for 2 h. Finally, we measured the absorbance of each well at a 450 nm wavelength.

### Colony formation assay

Five hundred cells were seeded in six-well plates and cultured at 37 °C. Clone size was observed daily until the majority of clones had more than 50 cells. Subsequently, the plates were stained with 0.2% crystalline violet solution for 30 min at room temperature and washed three times with PBS. These cells were then observed with a 4 × light microscope (77,002; Yuyan Instruments Co., Ltd. Shanghai, China) and photographed. The clone formation rate was calculated according to the following formula: clone formation rate (%) = (number of clones/500) * 100.

### Flow cytometric analysis of apoptosis

A total of 5 × 10^5^ cells were collected by centrifugation (250 × g, 5 min) at room temperature and washed 3 times with PBS. Cells were then resuspended in 100 µl binding buffer, followed by staining with 5 µl propidium iodide (PI) and Annexin-V for 20 min at room temperature protected from light. After staining, 400 µl of binding buffer was added to resuspend the samples. Apoptotic cells were subsequently assayed by flow cytometry (BD Biosciences) and visualized with FlowJo software (Ashland, OR, USA).

### Analysis of cell cycle distribution

A total of 1 × 10^6^ cells were collected by centrifugation at room temperature (250 × g, 5 min), fixed in 70% ethanol and incubated for 24 h at -20 °C. The cells were then stained with PI staining solution for 30 min at room temperature in the dark and then analyzed by flow cytometry (BD Biosciences). The percentage of cells in G1, S and G2/M phases was calculated with Modfit software (Version 5; Verity Software House, Inc.).

### Drug treatment

5-Fluorouracil (5-FU, S1209) and inhibitors of mTOR (rapamycin, S1039) were purchased from Selleck (Houston, TX, USA). The working concentration of 5-FU on HCT116 was 0–10 μg/ml. And the working concentration of 5-FU on SW620 was 0–20 μg/ml. The working concentration of rapamycin was 1 μM. For this assay, 1 × 10^4^ cells were seeded into 96-well plates and cultured with different concentrations of 5-FU for 24 h. And the cell viability was also measured by CCK-8, we detected OD450 at 24 h, OD450 of cells treating with 5-FU was normalized by OD450 of cells without treatment to get the cell viability (%). The cell viability was calculated according to the following formula: cell viability (%) = (OD450 of cells treating with 5-FU)/(OD450 of cells without treatment) * 100. The effect of compound treatment was confirmed by Western blotting.

### Subcutaneous xenografts of nude mice

All experimental procedures were approved by the Institutional Animal Care and Utilization Committee of the Animal Experimentation Center, Pudong, Fudan University. This study was performed according to the guidelines for reporting in vivo experimental studies in animals and the study is reported in accordance with ARRIVE guidelines. Animal breeding was charging by special person in the animal center. A total of 36 female Balb/c-nu mice weighing 20 ± 3 g, 5 weeks old, were purchased from Beijing Life River Laboratory Animal Technology Co., Ltd. and randomly divided into six groups: HCT116-NC, HCT116-KO1, HCT116-NC + 5-FU, HCT116-KO1 + 5-FU, HCT116-NC + rapamycin, and HCT116-KO1 + rapamycin (*n* = 6). A total of 5 × 10^6^ HCT116-KO1 or HCT116-NC cells were resuspended in 100 µl of PBS and injected into the axilla of mice. After one week, the long and short diameters of each subcutaneous tumor were measured every three days. Mice in both the HCT116-KO1 + 5-FU and HCT116-NC + 5-FU groups were injected intraperitoneally with 5-FU (20 mg/kg) every 2 days starting on Day 10. Mice in both the HCT116-NC + rapamycin and HCT116-KO1 + rapamycin groups were injected intraperitoneally with rapamycin (4 mg/kg) each day starting on Day 10. Tumor growth curves were plotted using the calculated tumor volumes (tumor volume = L x S2/2). All mice were sacrificed within one month of injection, and the subcutaneous tumors were completely excised. All mice were executed using the cervical dislocation method.

### Immunohistochemistry (IHC)

Protein expression levels in nude mouse subcutaneous tumors were measured by immunohistochemistry (IHC). Sections were heated, dewaxed, rehydrated and placed in sodium citrate buffer (pH = 6.0) for antigen repair. The slides were then immersed in 3% hydrogen peroxide to inhibit endogenous peroxidase activity. After 3 washes, the sections were incubated with primary antibody (rabbit anti-Ki67, 1:100 dilution, #ab15580, Abcam, Cambridge, UK; rabbit anti-p53, 1:100 dilution, # ab16665, Abcam, Cambridge, UK; rabbit anti-p53, 1:100 dilution, # ab16665, Abcam, Cambridge, UK; rabbit anti-mTOR, 1:100 dilution, # ab32028, Abcam, Cambridge, UK; rabbit anti-mTOR phospho S2448, 1:100 dilution, # ab109268, Abcam, Cambridge, UK; AKT1 rabbit mAb, 1:100 dilution, #A20799, Abclonal, Wuhan, China; Phospho-Akt Rabbit mAb, #AP1259, Abclonal, Wuhan, China;) overnight at 4 °C, followed by secondary antibody (anti-rabbit IgG, 1:2000 dilution, #7074; Cell Signaling, Danvers, MA, USA) at 37 °C for 40 min. After staining with 3,3-diaminobenzidine (DAB), slides were restained with Mayer's hematoxylin, dehydrated and sealed.

### TUNEL assay

Apoptotic cells in subcutaneous tumor tissue were determined by a TUNEL Apoptosis Assay Kit S711 (Millipore, USA) according to the protocol provided. Tumor tissues were fixed in 4% paraformaldehyde for 15 min. After dropwise addition of 0.1% Triton X-100 (freshly prepared from 0.1% sodium citrate), samples were incubated at room temperature for 15 min and then washed twice with PBS for 5 min. After TUNEL labeling, nuclei were restained with DAPI. TUNEL-positive apoptotic cells were counted and photographed using fluorescence microscopy.

### Statistical analysis

SPSS software (version 19.0, IBM Corp., Armonk, NY, USA) was used for statistical analysis of all the experimental data. GraphPad Prism (version 7, GraphPad Software, La Jolla, CA, USA) was used to determine the statistical results. All data are expressed as the mean ± standard deviation (mean ± SD). The statistical analysis of the data from 2 groups was performed using a t test. The comparisons of multiple groups were performed by one-way ANOVA and then an LSD-t test. *P* < 0.05 was considered significant [16].

## Results

### Knockout of MUC3A repressed the growth of CRC and promoted cell cycle arrest

**Fig. 1:**
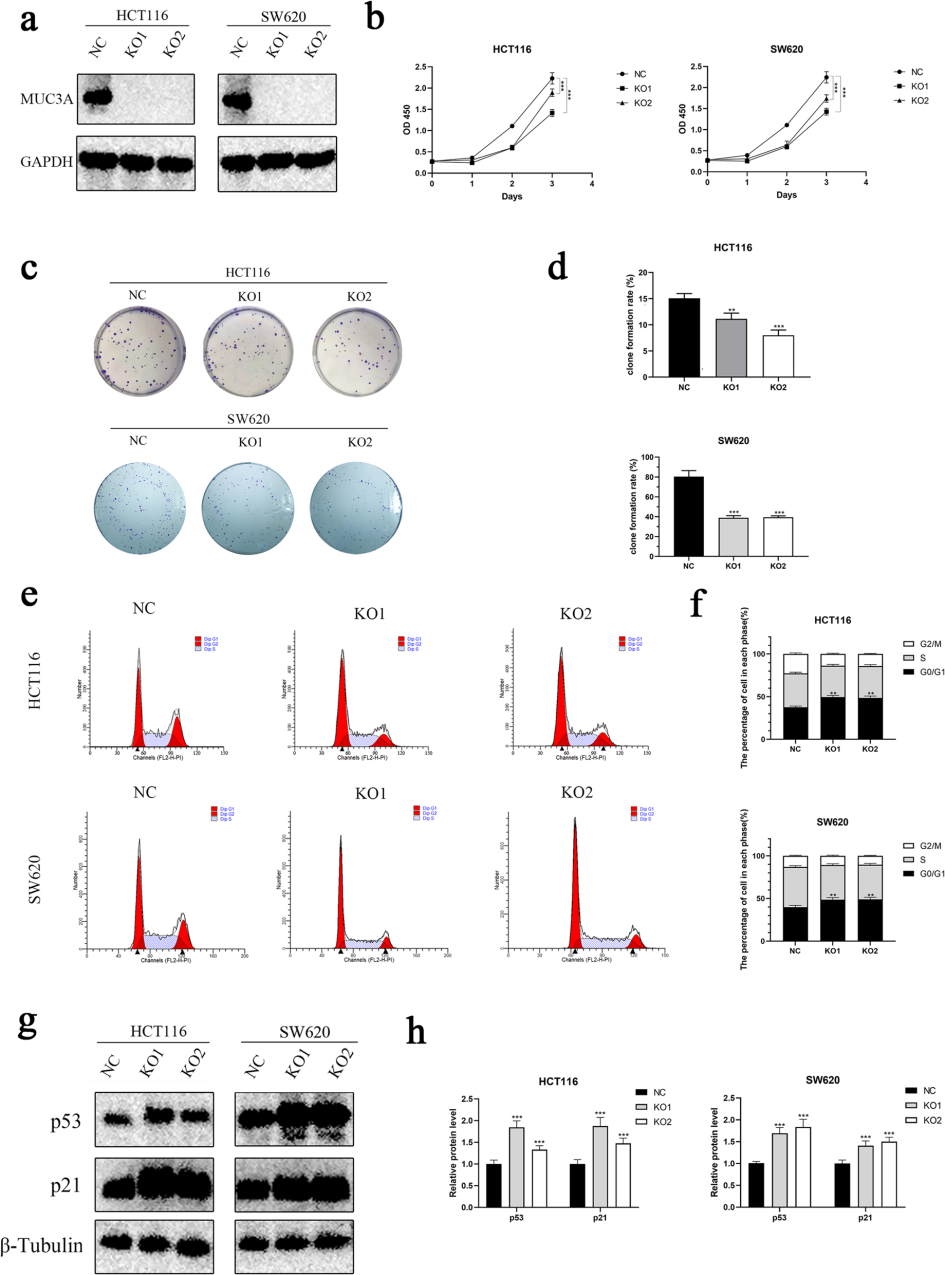
Knockout of MUC3A repressed the growth of CRC and promoted cell cycle arrest. **A** The expression of MUC3A of MUC3A knockout cells (KO1, KO2) and control cells (NC) by Western blotting analysis. **B** The proliferation of MUC3A knockout cells (KO1, KO2) and control cells (NC) determined by CCK-8 assay. **C D** Clone formation ability of CRC cells. **E F** Cell cycle of CRC cells detected by flow cytometry. **G H** Expression of p21 and p53 in CRC cells determined by Western blotting analysis. (**P < 0.01, ***P < 0.001)

MUC3A protein expression was significantly reduced in HCT116 and SW620 cells after MUC3A gene knockout (Fig. 1a). To determine the association between MUC3A and cell proliferation, CCK-8 and colony formation assays were performed. Knockout of MUC3A significantly repressed the proliferation ability of HCT116 and SW620 cells (Fig. 1b). Similar to the CCK-8 assay, the colony formation rate of HCT116 and SW620 cells was also inhibited by knockout of MUC3A (Fig. 1c, d). These results suggested that MUC3A may play an important role in the growth of CRC cells. As the cell cycle shows a close association with the proliferation of cells, we further measured the cell cycle of HCT116 and SW620 cells by flow cytometry (Fig. 1e). We found that knockout of MUC3A increased the percentage of G0/G1-phase cells and decreased the percentage of S-phase cells (Fig. 1f). These results suggested that knockout of MUC3A promotes G0/G1 phase arrest in CRC cells. We further detected cell cycle-related proteins and found that MUC3A knockout induced upregulation of p53 and p21 proteins, which are important regulators of the cell cycle (Fig. 1g, h).

### Knockout of MUC3A promoted the sensitivity to chemotherapy and apoptosis of CRC

**Fig. 2:**
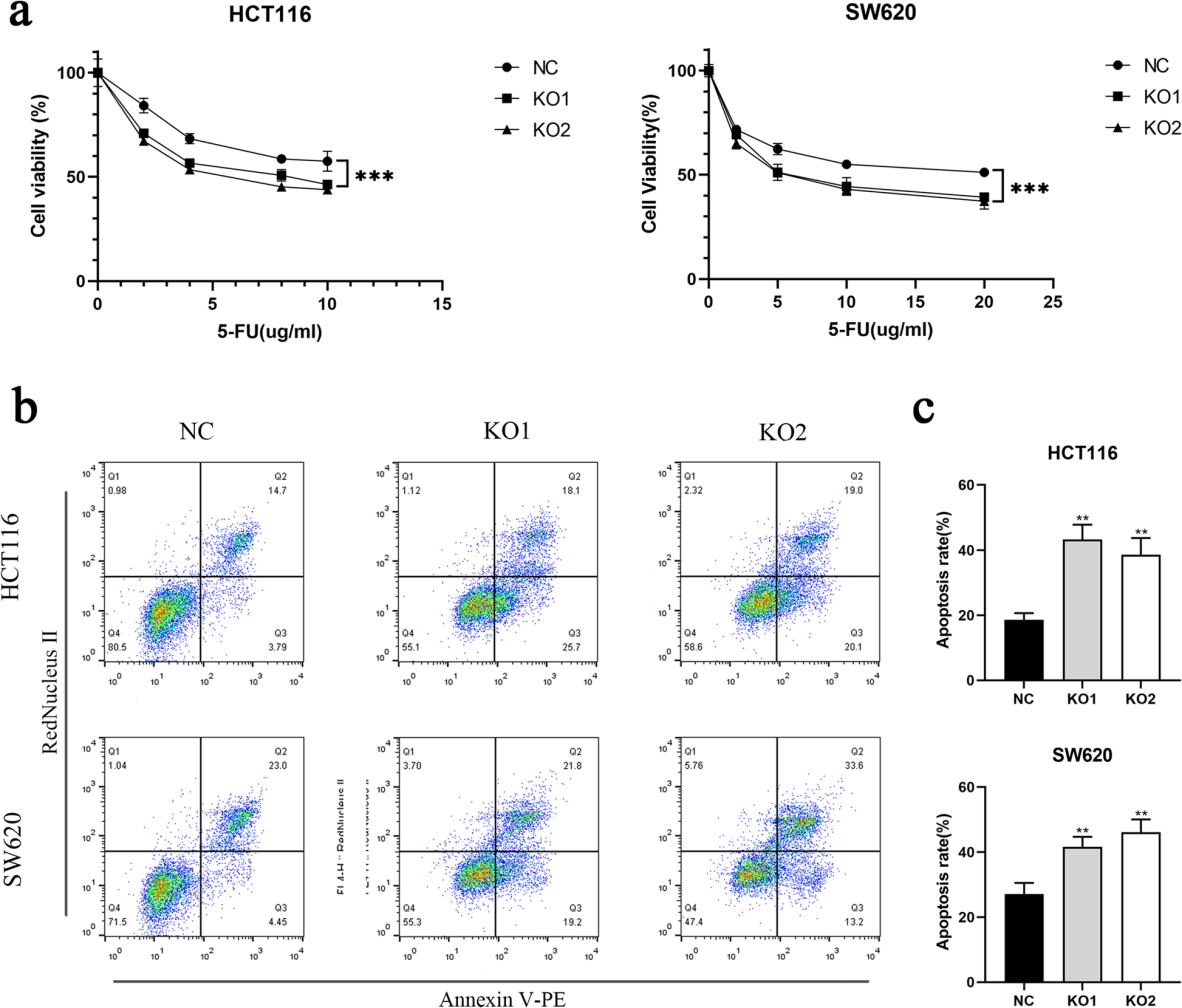
Knockout of MUC3A promoted sensitivity to chemotherapy and apoptosis of CRC. **A** Cell viability of CRC cells treated with multiple doses of 5-FU (0–20 µg/ml, 24 h). **B C** Apoptosis rate of CRC cells treated with 10 µg/ml 5-FU for 24 h. (**P < 0.01, ***P < 0.001)

5-Fluorouracil is the main chemotherapeutic agent commonly applied in the treatment of CRC patients. Therefore, we further investigated whether MUC3A affects the response to 5-FU in HCT116 and SW620 cells. We found that MUC3A knockout cells were more sensitive to 5-FU (Fig. 2a). Then, we examined the apoptosis rate of cells under 5-FU treatment (Fig. 2b) and found that MUC3A knockout cell lines had more cells undergoing apoptosis (Fig. 2c).

### Knockout of MUC3A repressed the migration and invasion abilities of CRC

**Fig. 3:**
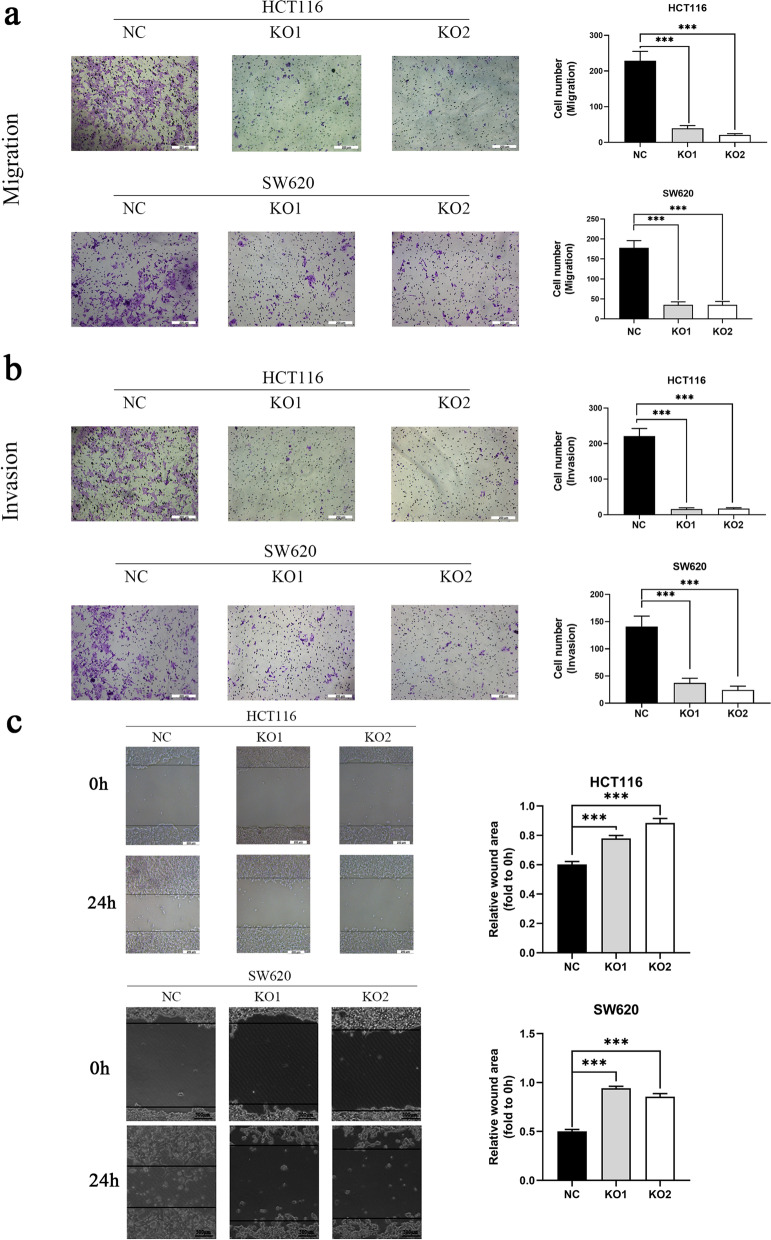
Knockout of MUC3A repressed the migration and invasion abilities of CRC. **A B** Migration and invasion abilities of CRC cells determined by Transwell assays. **C** Migration ability of CRC cells detected by wound healing assay. (***P < 0.001)

To investigate the association between MUC3A and cell invasion and migration abilities, Transwell and wound healing assays were performed. Knockout of MUC3A significantly repressed both the invasion and migration abilities of HCT116 and SW620 cells (Fig. 3a, b). Similarly, in the wound healing assay, the migration ability of MUC3A knockout cells was also significantly reduced compared with that of control cells (Fig. 3c).

### Knockout of MUC3A downregulated the PI3K/AKT/mTOR signaling pathway in CRC

**Fig. 4:**
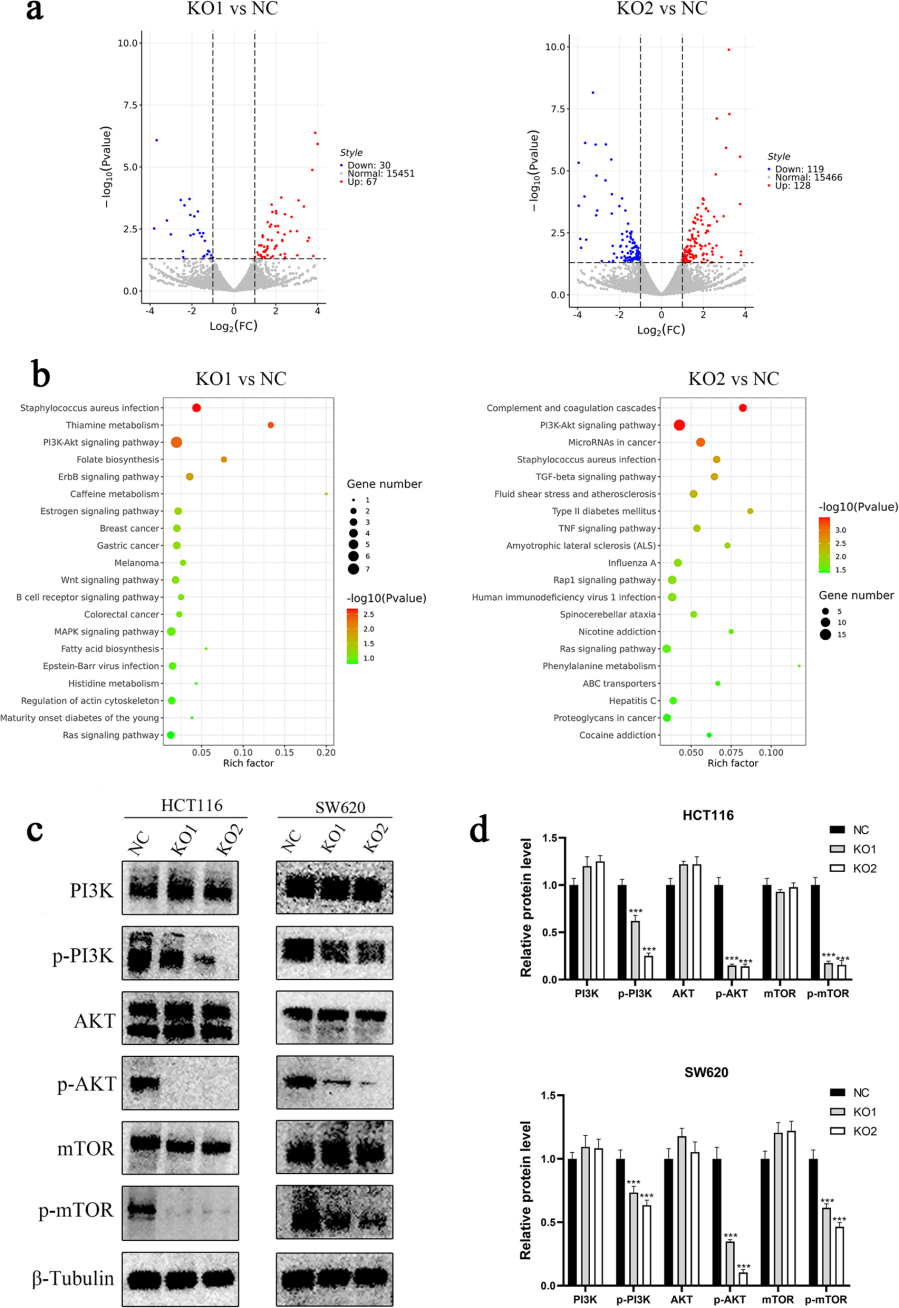
Knockout of MUC3A downregulated the PI3K/AKT/mTOR signaling pathway in CRC. **A B** Volcano map visualized by R software revealing differentially expressed genes (DEGs) between MUC3A knockout cells and control cells. B Pathway enrichment analysis of DEGs analyzed and visualized by R software. **C D** Validation of inactivation of PI3K/Akt/mTOR pathways in MUC3A knockout cells performed by Western blotting analysis. (**P < 0.01, ***P < 0.001)

To further investigate the mechanism of MUC3A-regulated progression in CRC, we performed RNA-seq and identified differentially expressed genes (DEGs) between MUC3A knockout and control cell lines (Fig. 4a). Through enrichment analysis of the signaling pathway of these DEGs, we found that MUC3A was closely associated with the PI3K/AKT signaling pathway (Fig. 4b). The datasets generated and analysed during the current study are available in the GEO repository (record GSE201107). Subsequently, we further validated that knockout of MUC3A repressed the PI3K/AKT/mTOR signaling pathway by Western blot analysis (Fig. 4c, d).

### MUC3A regulated the growth and cell cycle of CRC through the PI3K/AKT/mTOR signaling pathways

**Fig. 5:**
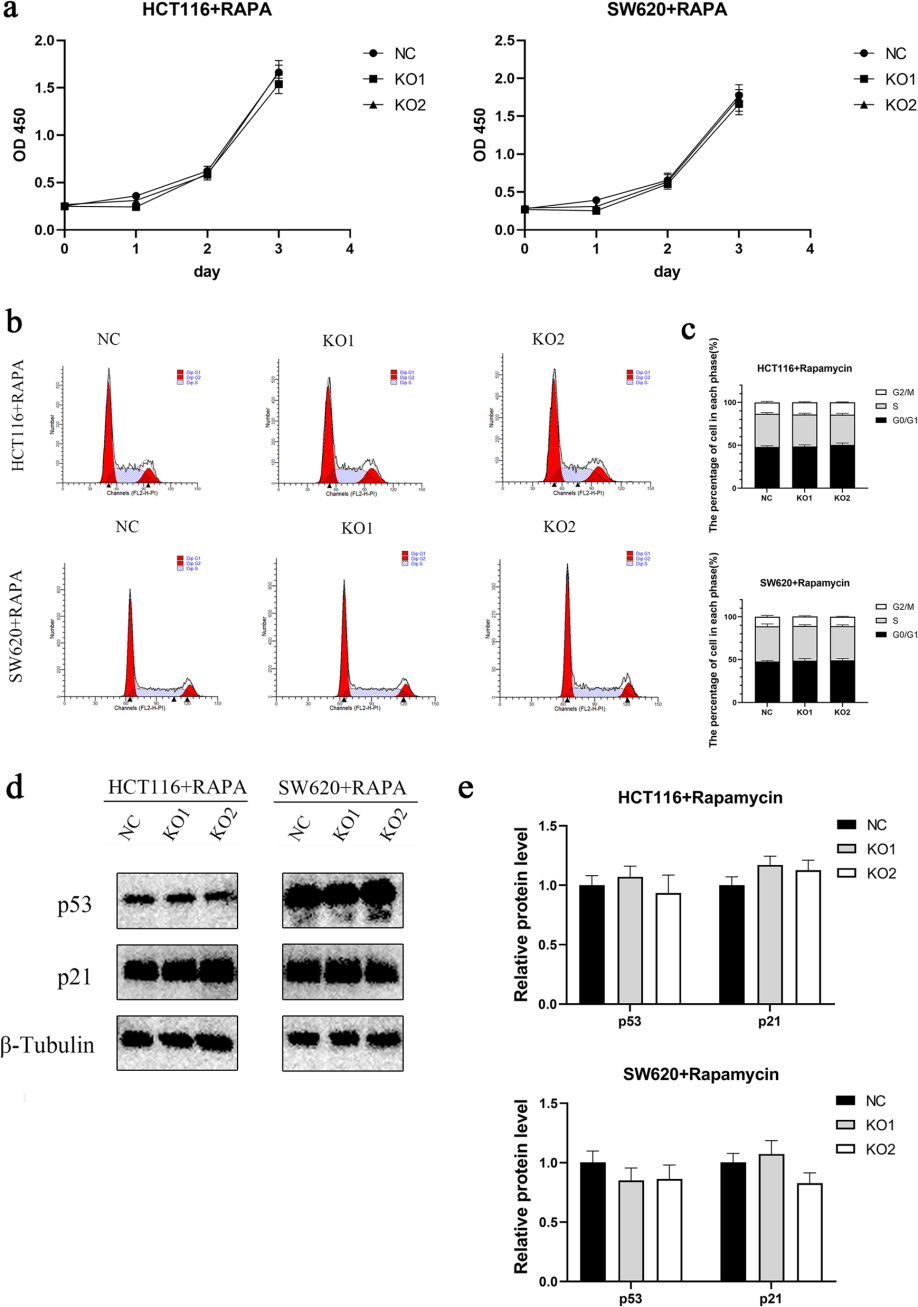
MUC3A regulated the growth and cell cycle of CRC and through the PI3K/AKT/mTOR signaling pathway.** A** The proliferation of MUC3A knockout cells (KO1, KO2) and control cells (NC) treated with the mTOR inhibitor rapamycin (1 µM for 4 h). **B C** Cell cycle of CRC cells treated with rapamycin. **D E** Expression of p21 and p53 in CRC cells treated with rapamycin

The significant difference in proliferation ability that existed between the MUC3A knockout cell line and control cells was lost with rapamycin intervention, the mTOR inhibitor (Fig. 5a). The corresponding differences in the cell cycle (Fig. 5b, c) and differences between cycle-related proteins (Fig. 5d, e) were also eliminated. These findings suggest that MUC3A regulated the proliferation and cell cycle of CRC cells through the PI3K/AKT/mTOR signaling pathway.

### MUC3A promoted the migration and invasion of CRC through the PI3K/AKT/mTOR signaling pathway

**Fig. 6:**
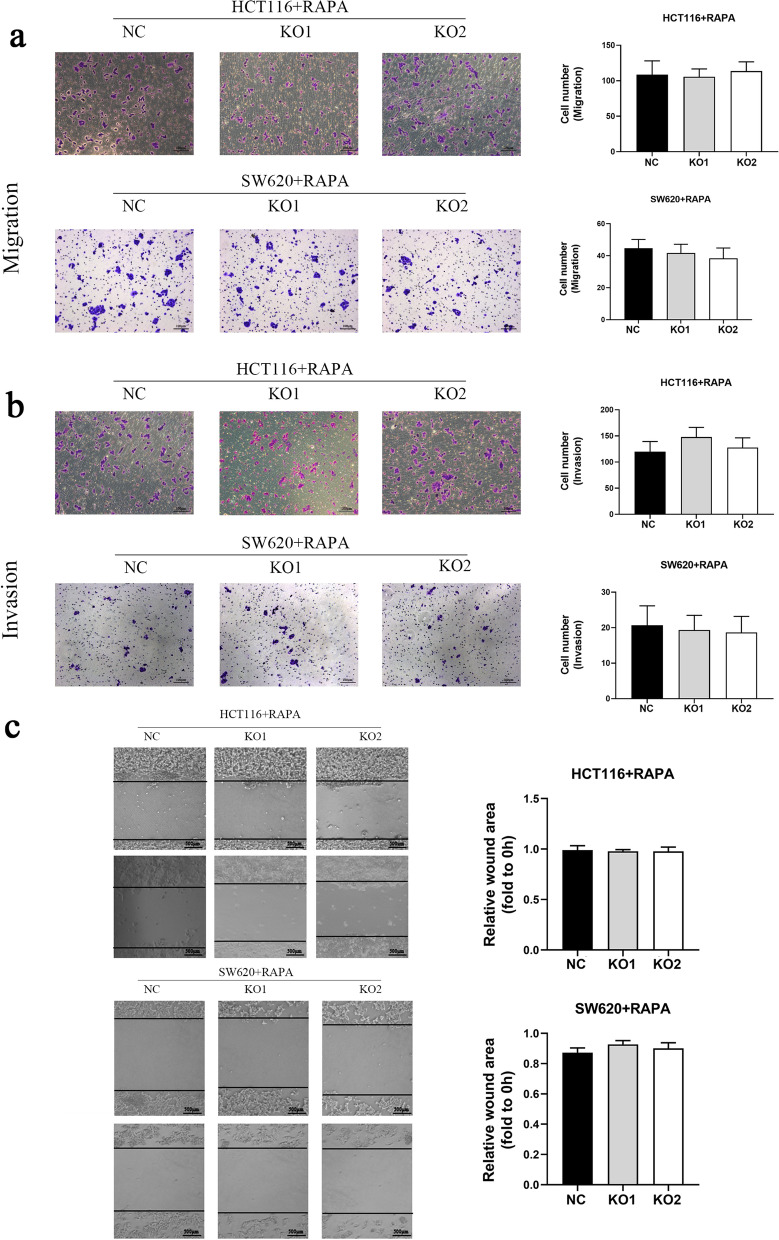
MUC3A promoted the migration and invasion of CRC through the PI3K/AKT/mTOR signaling pathway. **A B** Migration and invasion abilities of CRC cells treated with rapamycin (1 µM for 4 h). **C** Migration ability of CRC cells treated with rapamycin detected by wound healing assay (1 µM for 4 h)

Similarly, the significant differences in migration and invasion ability that existed between MUC3A knockout cell lines and controls were eliminated with rapamycin intervention (Fig. 6a, b, c). This elimination confirmed that MUC3A promotes CRC cell metastasis and acts through the PI3K/AKT/mTOR signaling pathway.

### Knockout of MUC3A repressed cell growth in vivo

**Fig. 7:**
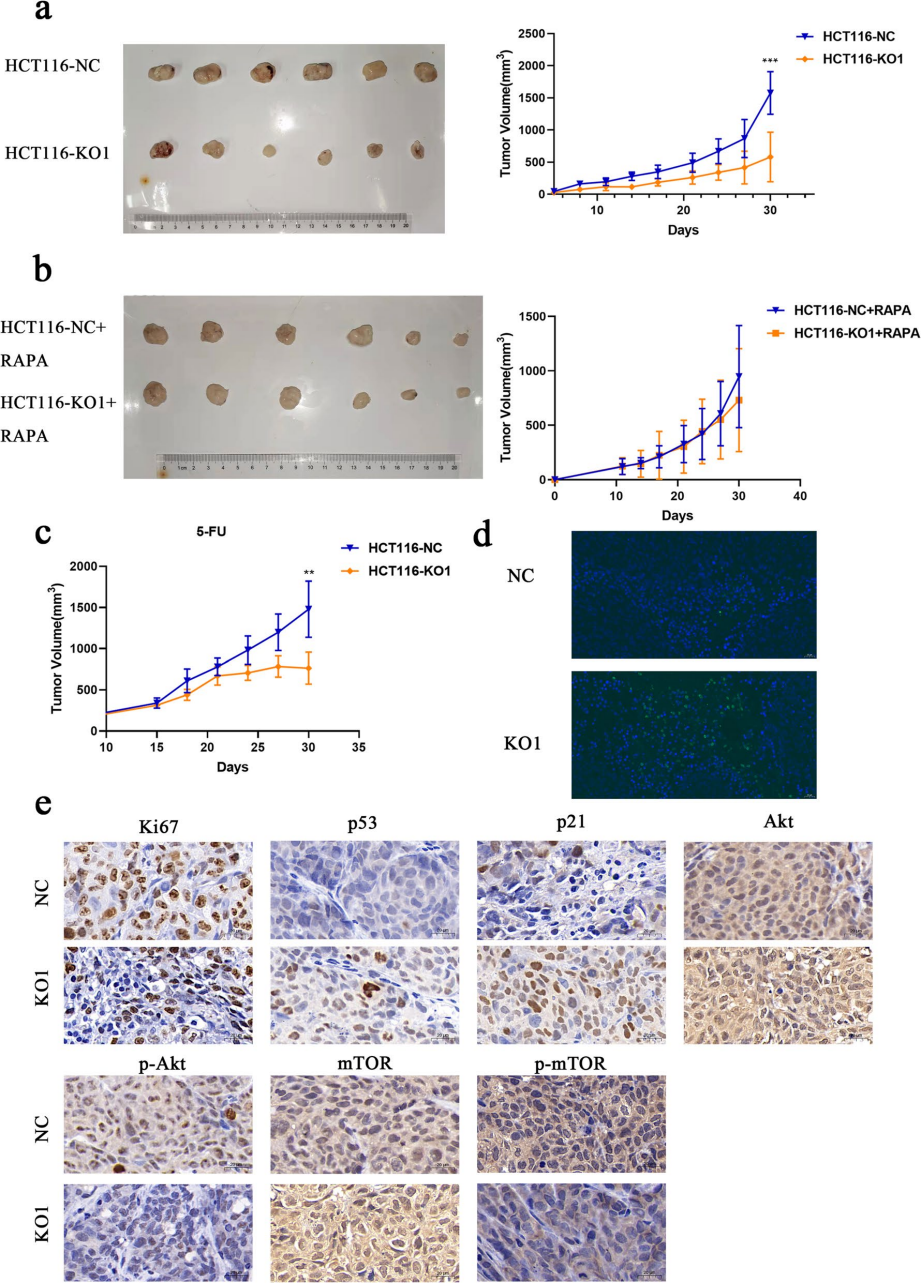
Knockout of MUC3A repressed cell growth in vivo. **A-C** The growth curve of CRC cells in vivo. **A** Without any treatment. **B** With rapamycin treatment (4 mg/kg, per day). **C** With 5-FU treatment (20 mg/kg, per 2 days). **D** Apoptosis rate of xenografts detected by TUNEL staining. E Expression of Ki67, p53, p21 and PI3K/Akt/mTOR pathway-associated proteins detected by IHC (**P < 0.01, ***P < 0.001)

To study the role of MUC3A in CRC in vivo, a xenograft model was constructed. The growth of the HCT116-KO1 group was significantly slower than that of the control group from 10 to 30 days (Fig. 7a). However, the difference was eliminated with rapamycin treatment (Fig. 7b). In contrast, the same significant difference was observed between the two groups of mice treated with 5-FU (Fig. 7c), and the tumor volume of the HCT116-KO1 + 5-FU group was significantly smaller than that of the HCT116-NC + 5-FU group. By immunofluorescence detection of TUNEL staining, we observed more apoptotic cells in MUC3A knockout subcutaneous tumors (Fig. 7d). Consistent with cellular experiments, knockout of MUC3A also repressed Ki67 (the proliferation index), p-AKT, and p-mTOR but promoted p21 and p53 proteins (Fig. 7e).

## Discussion

The incidence of colorectal cancer is next to lung cancer and gastric carcinoma. And it ranks second as a cause of death due to cancer in the world [1]. It is estimated that up to two-thirds of CRC can be attributed to major lifestyle and modifiable risk factors [17]. A number of studies have demonstrated that high intakes of red meat and processed meat increase the risk of colorectal cancer [18]. The five-year relative survival rate for people whose colorectal cancer is treated in an early stage is greater than 90 percent [19]. However, colorectal cancer can also exist for years, hardly creating symptoms until its most virulent stage and patients survival over 5 years is only about 20% [19]. Therefore, the mechanism of the progression of colorectal cancer and new therapeutic targets are urgently needed to pay attention to.

MUC3A encodes the MUC3A protein, a highly glycosylated membrane-associated mucin. In previous studies, overexpression of MUC3A in different types of cancers showed a close association with metastasis and poor prognosis [11–14, 20]. MUC3A is highly expressed in non-small cell lung cancer and promotes its progression by activating the NFκB pathway and attenuating radiosensitivity [15]. However, few studies have focused on the role of MUC3A in CRC. Our research found that knockout of MUC3A repressed the proliferation ability of CRC cells by p21- and p53-regulated cell cycle arrest. p21 is a member of the Clp family, which is a cyclin-dependent kinase inhibitor located downstream of p53. p21 and p53 can together form the G1 checkpoint of the cell cycle, thus regulating cell cycle arrest [21, 22]. Additionally, we also found that knockout of MUC3A repressed the chemoresistance and invasion ability of CRC cells, whereas related mechanisms remain unclear. Therefore, we performed RNA-seq to identify DEGs between MUC3A knockout cells and control cells. We found that these DEGs showed a close association with the PI3K/AKT signaling pathway. These data provide important directions for us to further explore the potential mechanisms involved in the role of MUC3A in CRC cells.

A growing number of studies have reported that the PI3K/AKT signaling pathway plays an important role in regulating the cell proliferation, growth, invasion, metabolism and motility of cancers [23]. AKT can regulate the tumor cell cycle by phosphorylating cell cycle-related proteins such as p21 [24]. Activated AKT inhibits apoptosis by phosphorylating substrates, including BAD (BCL-2/Bcl-XL antagonist, causing cell death), glycogen synthase kinase-3, Forkhead transcription factor, and nitric oxide synthase, inhibiting apoptosis through various mechanisms [25]. Mammalian target of rapamycin (mTOR) regulates protein synthesis, cell growth, metabolism, senescence, regeneration, autophagy, and many other fundamental cellular activities and is dysregulated in many human tumors [26]. Previous studies have shown that activation of mTOR promotes tumor growth and metastasis. Many mTOR inhibitors have been developed to treat cancer [27]. Among them, rapamycin is the most promising and has been widely applied in the clinic [28].

We observed significant inhibition of the PI3K/Akt/mTOR pathway in MUC3A knockout cells. Interestingly, after treatment with the mTOR inhibitor rapamycin, the differences in proliferation, cell cycle arrest, and invasion abilities were eliminated between MUC3A knockout cells and control cells. Thus, our data confirmed that MUC3A promotes the progression of CRC cells by activating PI3K/Akt/mTOR. Meanwhile, we further suggested mTOR as a promising treatment for CRC patients with high MUC3A expression. And MUC3A may be a new and tissue-specific therapeutic target for CRC, while abnormal activation of the Akt signaling pathway is common in CRC.

## Conclusions

In conclusion, knockout of MUC3A inhibited tumor proliferation, invasion, and chemoresistance in CRC via the PI3k/Akt/mTOR signaling pathway. MUC3A is an oncogene in colorectal cancer. And MUC3A serves as potential targets for the treatment of CRC, especially those with abnormal activation of PI3K/Akt/mTOR signaling pathway.

## Supplementary Information

**Additional file 1.**

**Additional file 2.**

## Data Availability

The datasets used and analyzed during the current study are available from the corresponding author on reasonable request. And the datasets generated and analysed during the current study are available in the GEO repository (record GSE201107). The following secure token has been created to allow review of record GSE201107 while it remains in private status: mzgrekwqntmvjub.
