# Supplementary figures and images for: MUC3A promotes the progression of colorectal cancer through the PI3K/Akt/mTOR pathway
Source: BMC Cancer. 2022 Jun 2;22:602. doi: 10.1186/s12885-022-09709-8 (PMC9161576; doi:10.1186/s12885-022-09709-8)

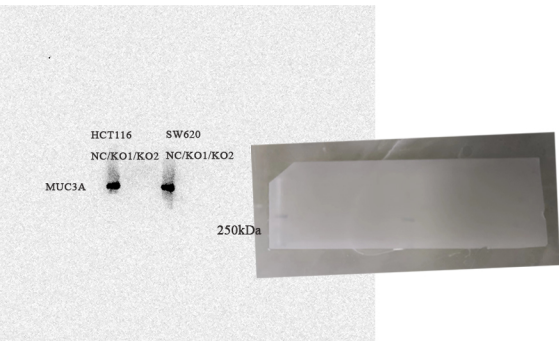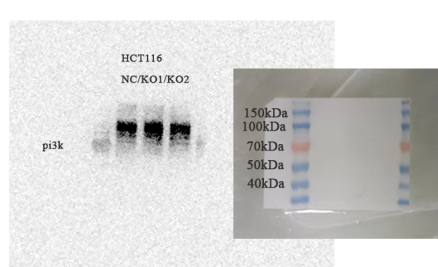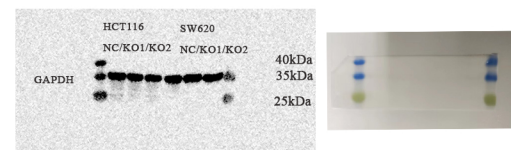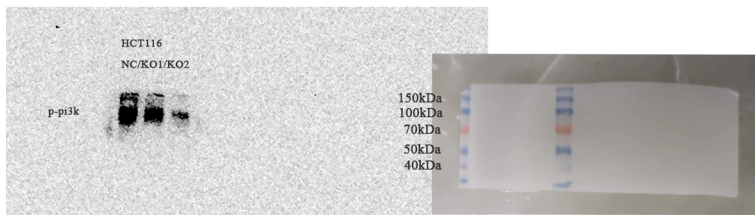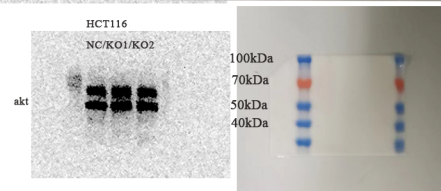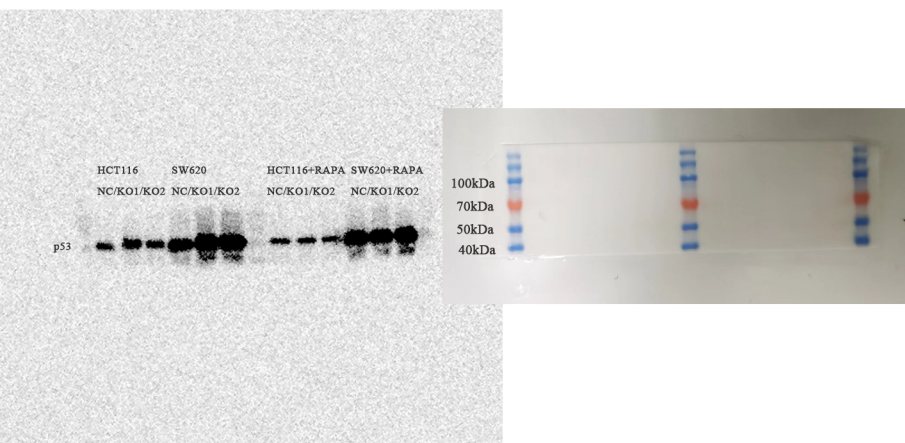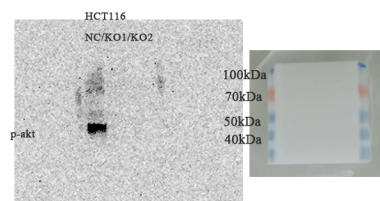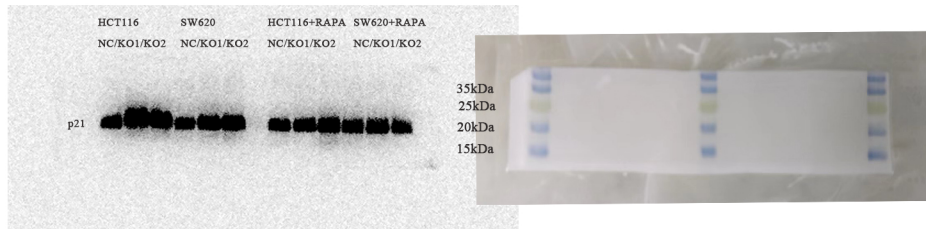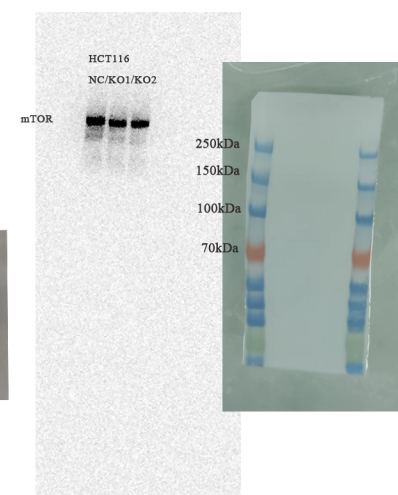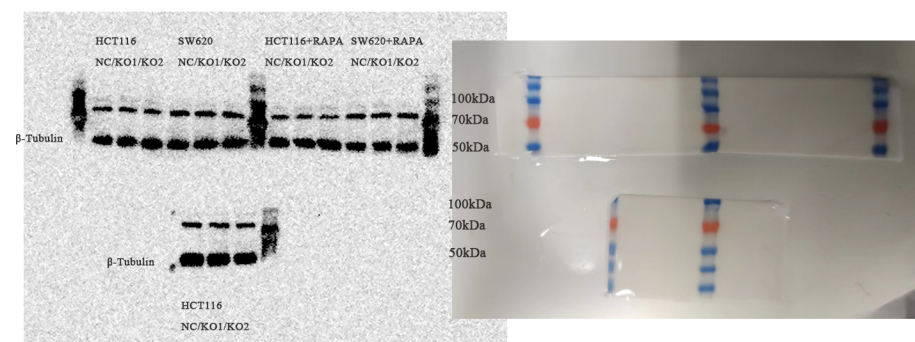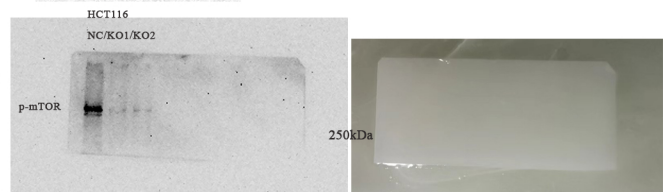

Supplement: Supplementary file 1 — Additional file 1. [file 12885_2022_9709_MOESM1_ESM.pdf]
