# Supplementary material for: MUC3A promotes the progression of colorectal cancer through the PI3K/Akt/mTOR pathway
Source: BMC Cancer. 2022 Jun 2;22:602. doi: 10.1186/s12885-022-09709-8 (PMC9161576; doi:10.1186/s12885-022-09709-8)
Supplement: Supplementary file 2 — Additional file 2. [file 12885_2022_9709_MOESM2_ESM.pdf]

The datasets generated and analysed during the current study are available in the GEO repository (record GSE201107).

The following secure token has been created to allow review of record GSE201107 while it remains in private status: **mzgrekwqntmvjub**
